# Heavy water induces bundling in entangled actin networks†

**DOI:** 10.1039/d3ra03917j

**Published:** 2023-08-18

**Authors:** Paul Mollenkopf, Dusan Prascevic, Thomas M. Bayerl, Josef A. Käs, Jörg Schnauß

**Affiliations:** a Department of Physiology, University of Pennsylvania Philadelphia PA 19104 USA; b Peter-Debye Institute for Soft Matter Physics, Leipzig University 04103 Leipzig Germany joerg.schnauss@uni-leipzig.de; c Inventages 16 Northfields Prospect Business Centre, Putney Bridge Rd London SW181PE UK; d Fraunhofer Institute for Cell Therapy and Immunology 04103 Leipzig Germany; e Unconventional Computing Lab, Department of Computer Science and Creative Technologies, University of the West of England Bristol BS16 1QY UK

## Abstract

Heavy water is known to affect many different biological systems, with the most striking effects observed at the cellular level. Many dynamic processes, such as migration or invasion, but also central processes of cell proliferation are measurably inhibited by the presence of deuterium oxide (D_2_O). Furthermore, individual cell deformabilities are significantly decreased upon D_2_O treatment. In order to understand the origin of these effects, we studied entangled filamentous actin networks, a commonly used model system for the cytoskeleton, which is considered a central functional element for dynamic cellular processes. Using bulk shear rheology to extract rheological signatures of reconstituted actin networks at varying concentrations of D_2_O, we found a non-monotonic behavior, which is explainable by a drastic change in the actin network architecture. Applying light scattering and fluorescence microscopy, we were able to demonstrate that the presence of deuterium oxide induces bundling in reconstituted entangled networks of filamentous actin. This constitutes an entirely novel and previously undescribed actin bundling mechanism.

## Abstract 2

In the presence of deuterium oxide actin filaments in entangled networks align to form thick bundles.

## Graphical abstract

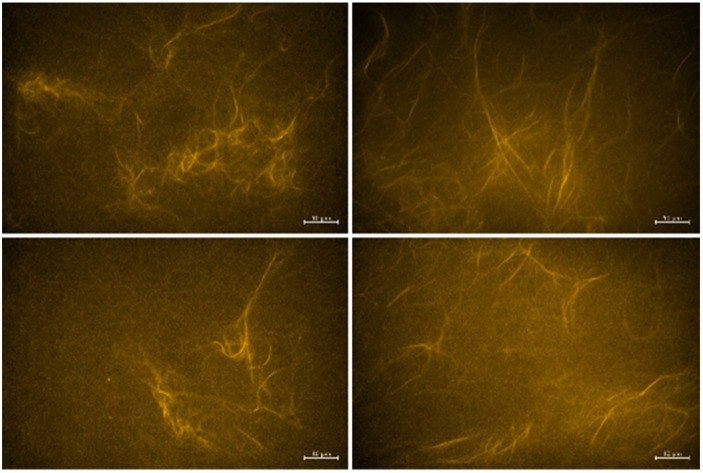

## I. Introduction

Deuterium oxide (D_2_O), generally known as heavy water, surprises with fascinating properties that differ from those of conventional water. In D_2_O, the ordinary hydrogen (protium) atoms are replaced by the hydrogen isotope deuterium, which in contrast to ordinary hydrogen comprises an additional neutron. As a consequence, deuterium atoms are roughly twice as heavy as protium atoms, resulting in different chemical and physical properties. Hence, D_2_O is distinctly heavier than H_2_O and exhibits an 11% higher density and a 23% higher viscosity.^1^ Even though the disruptive effects of heavy water on physiological processes such as the cell cycle and circadian rhythm have been known for decades, the exact mechanism behind many of these effects remains elusive.^2–4^ In recent years, heavy water experienced a renaissance in scientific interest, with more and more studies trying to unravel the specific manner in which cellular structures are affected by heavy water. In a recent study, Schnauß *et al.* showed that, by exchanging hydrogen bonds with stronger deuterium bonds, protein–solvent interactions are significantly altered in the presence of D2O.^5^ This has had verifiably severe consequences for cellular dynamics, while cell morphology and phenotype remained unchanged. Cell proliferation as well as migration were reportedly slowed down in a reversible manner. The cause for this retardation of dynamics was experimentally identified in the D_2_O induced alteration of cell resistance *via* deformability measurements on individual, isolated cells. To better understand the origin of the changed cellular viscoelasticity, we study the impact of D_2_O on cytoskeletal properties using *in vitro* reconstituted entangled filamentous actin (F-actin) networks, an extensively studied polymer system.^6–9^ We employed bulk shear rheology to monitor the changing viscoelastic properties of entangled F-actin networks in the presence of heavy water, measuring in a concentration range between 0% D_2_O and 70% D_2_O in 10% increments. This investigation revealed a non-monotonic viscoelastic response for increasing heavy water content, similarly to what was reported by Schnauß *et al.*^5^ In addition to this, we used static light scattering (SLS) and fluorescence microscopy to observe the effect of heavy water on the morphology of F-actin networks. SLS revealed consistently increased scattering intensities for increasing heavy water content, indicating the presence of multi-filament formations. Fluorescence microscopy was further used to validate this finding by directly observing the decreasing isotropy of the networks, and eventual the formation of bundle structures for sufficiently high heavy water concentrations. The formation of bundle structures and the accompanying de-percolation of the networks can explain the observed non-monotonic trend of the networks' viscoelastic response with increasing heavy water content. A similar effect was previously reported for bundling induced by the addition of synthetic crosslinkers to *in vitro* F-actin networks.^10^ The discovery of this novel bundling mechanism also accounts for another puzzling observation reported by Schnauß *et al.*, namely the non-monotonic trend of the stickiness parameter used to quantify the strength of inter-filament interactions within the network.^5^ In conclusion, heavy water-induced bundling presents a completely novel and unexpected bundling mechanism for actin which accounts for previously unexplainable observations in the rheology of actin networks.

## II. Results

### A Rheology

**Fig. 1:**
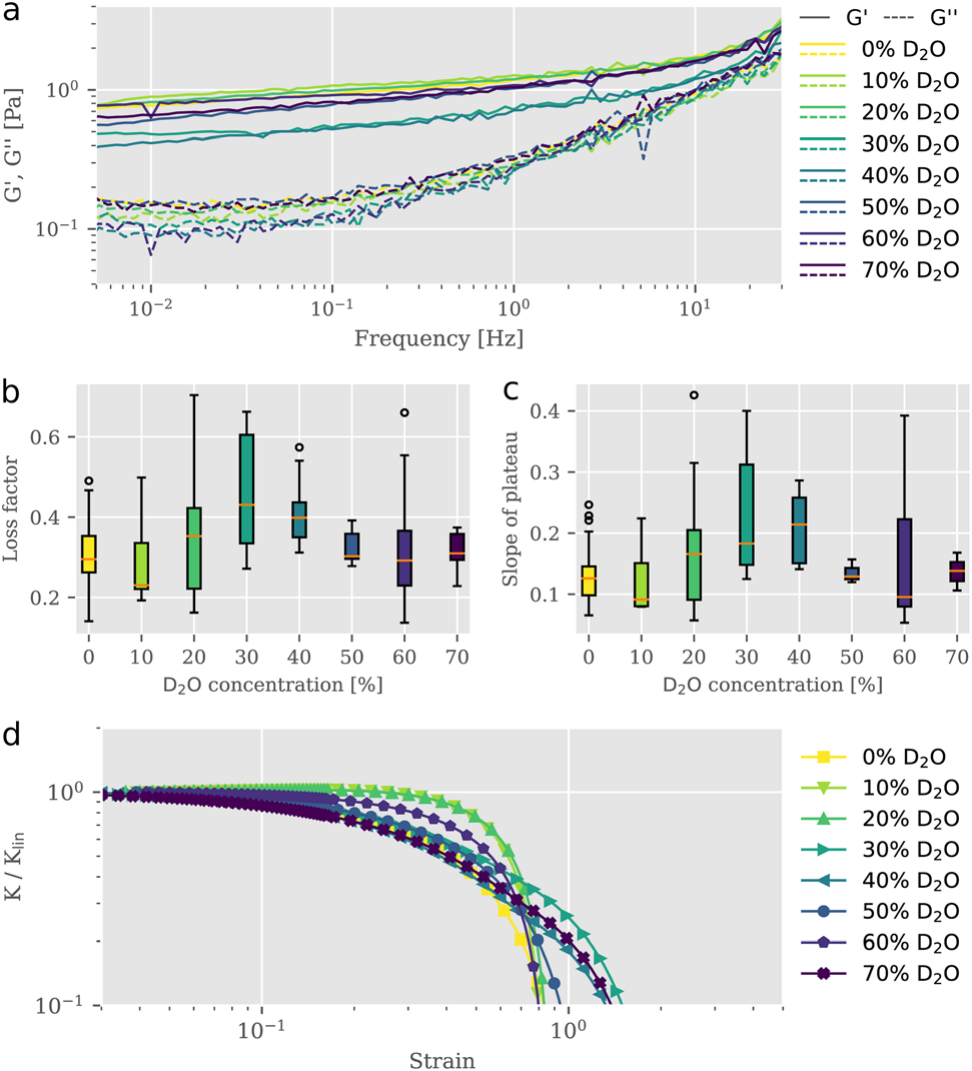
Rheological characterization of F-actin networks in varying presence of heavy water. (a) Elastic and loss moduli (*G*′, *G*′′) of F-actin networks for increasing heavy water solvent content. Initially, the magnitude of the elastic modulus seems to increase slightly for 10%- and 20% D_2_O concentrations. The value then drops to a minimum for 30%- and 40% D_2_O concentrations, before rebounding somewhat for the remaining heavy water concentrations (50%-, 60%- and 70% D_2_O). (b) The loss factor, here defined as the tan *δ* value at 1 Hz, is often used to illustratively represent the changing viscoelastic nature of materials. Medians are indicated by orange lines, with boxplot whiskers marking the ±1.5× interquartile range. Outliers are indicated as empty circles above and below whiskers. Following a similar trend as the slopes of the elastic modulus, the loss factor initially increases with an apparent maximum at 30% D_2_O. For values above and including 50% D_2_O, the loss factor is comparable to the control measurement. (c) Slope of the elastic modulus *G*′ is determined in the range of 0.01 Hz to 10 Hz and is used to characterize changing interactions within the network. An initial trend of increasing slopes for heavy water concentrations up to 40% D_2_O was observed, with subsequent rebound to values similar to control sample. (d) Differential shear modulus *K* normalized with its value from the linear regime *K*_lin_ as a function of applied strain on the sample. We observed no strain-stiffening in the control measurement nor in any of the D_2_O treated networks. Increasing the D_2_O content resulted in a non-monotonic response, similar to what was observed in the linear rheology, albeit with relatively minor differences between the samples.

To study the impact of D_2_O on actin structures and their mechanical properties, we employed bulk shear rheology on reconstituted F-actin networks at a concentration of 0.5 mg ml^−1^ suspended in buffer solutions of varying D_2_O content. Samples were prepared at identical conditions but with differing D_2_O concentrations, where the total volume of water (H_2_O + D_2_O) remained the same. For each sample, we measured the linear viscoelastic behavior (Fig. 1a) and extracted the parameters shown in Fig. 1b and c, prior to exposing it to strains in the nonlinear regime to test the behavior for large deformations (Fig. 1d). We tested the frequency dependent complex modulus G* for D_2_O concentrations between 0% and 70% in a frequency range from 0.01 Hz to 30 Hz. To exclude possible effects that may arise from an alteration of mixing dynamics due to the physical properties of D_2_O,^11^ great care was taken to ensure proper mixing. From the storage and the loss moduli illustrated in Fig. 1a, we derived the loss factor tan *δ*, defined as tan *δ* = *G*′′/*G*′ at 1 Hz (Fig. 1b), as well as the slopes of the elastic storage moduli (Fig. 1c). Interestingly, we observed a non-monotonic behavior of loss factors and slopes of the elastic plateau with stepwise increasing D_2_O concentration. For 10% D_2_O content tan *δ* is lowered compared to the value for the reference taken in the absence of D_2_O. A rheological signature of entangled F-actin solutions is a weak power-law behavior, expressed in a small apparent slope of *G*′ in a double logarithmic plot. Accompanied by the increase in elasticity, we found a reduced frequency dependency of the storage modulus. This behavior is associated with increasing attractive interactions between individual filaments in an entangled network and a consequential decrease of relaxation dynamics, as described previously.^12,13^ Between concentrations of 10% and 30% D_2_O the loss factor gradually increased to reach its maximum mean value of 0.45 which was roughly 1.5-fold the initial mean value. The slope of the elastic plateau increased for concentrations higher than 10% to reach its maximum mean value of 0.22 at 30% D_2_O. Networks in solutions exceeding 40% D_2_O revealed slope and loss factor values that were decreasing. The evaluation of the linear rheometry data clearly indicated variations of the viscoelastic properties of F-actin networks as a result of the D_2_O treatment. However, the signatures of the change in the derived parameters, the loss factor tan *δ* and the slope of the plateau, were not unique. We found that networks, exposed to gradually increasing presence of D_2_O, expressed viscoelastic properties which altered between stiffening and softening behavior in a non-monotonic fashion, resembling the characteristics of physically crosslinked networks.^10^ The non-monotonic trend of viscoelastic parameters under linear deformations, more precisely the increased values for loss factor and slope of plateau at intermediate D_2_O concentrations and their subsequent rebound to values similarly to the control, also transferred to the networks' responses in the nonlinear strain regime. This response is quantified with the differential modulus *K*, defined as the local derivative of stress *σ* over strain *γ* as described by Semmrich *et al.*^13,14^ We exposed the networks to strains increasing with a constant rate up to deformations beyond the network's fracture point. We observed essentially no strain-stiffening for normal water control conditions, in line with previous studies.^15,16^ The addition of D_2_O did not significantly affect this behavior, with only slight differences for different D_2_O concentration (Fig. 1d). Plotting the differential shear modulus *K* normalized with its value from the linear regime *K*_lin_ as a function of stress *σ* allowed for the evaluation of the stress value at which the network started to yield, known as the yield stress. Similar to the parameters extracted from the linear rheology, the yield stresses also exhibited non-monotonic behavior with increasing D_2_O content (ESI Fig. S1†). The non-monotonic signatures in the linear as well as in the nonlinear rheology indicated that the presence of D_2_O induced structural changes within the network architecture, drastically affecting their mechanical appearance. A similar triphasic behavior emerging from structural polymorphism due to the effect of specific crosslinkers added to F-actin networks was reported previously by Lorenz *et al.* They observed that crosslinker-to-actin concentration ratios 0.01 < *R* < 0.08 led to local anisotropies in the form of bundles and reduced the effective actin concentration in the percolated background, thereby weakening the overall structure resulting in a lowered *G*′.^10^ These crosslinker-to-actin concentration ratios defined a coexistence regime, in which bundle structures could be found alongside a mostly isotropic network of filaments. Increasing the crosslinker concentration beyond this concentration ratio resulted in the formation of bundle networks, which were characterized with a monotonically increasing stiffness. In this respect, the non-monotonic mechanical response of the networks that we observed provides indirect evidence of a bundle formation corresponding to this coexistence regime. Increasing loss factor values for the intermediate D_2_O concentration regime suggest a decrease in elasticity by an increase of the effective mesh size due to the local concentration of filaments into bundles. Further increasing the D_2_O concentration leads to thicker bundles, which can compensate for the increased in-homogeneity with higher bending moduli, resulting in higher elasticities.

### B Static light scattering

**Fig. 2:**
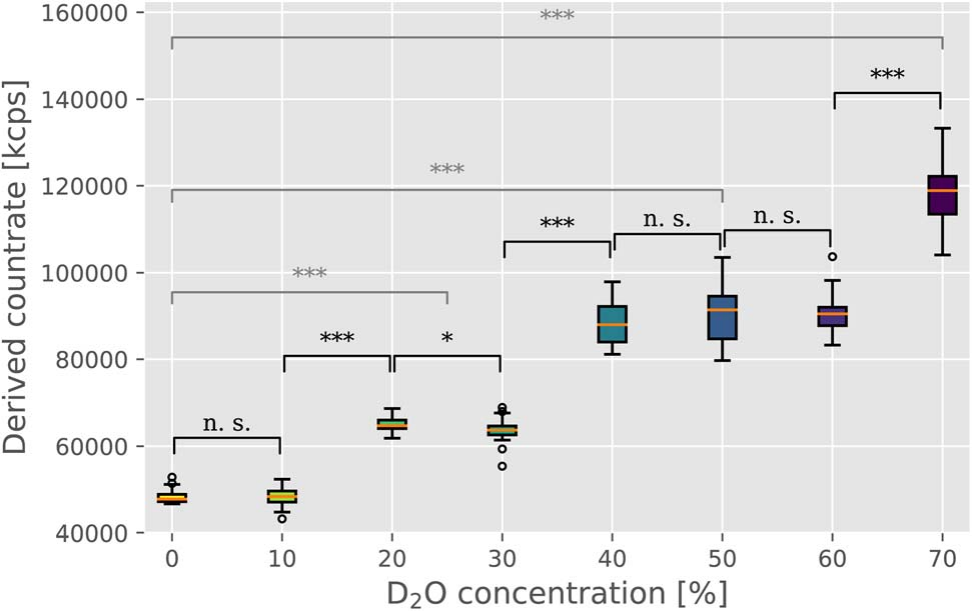
Static light scattering was used to evaluate the changing morphology of F-actin networks in varying heavy water conditions. Derived count rate, here expressed in kilo counts per second (kcps), provides a direct measure of the scattering intensity, which has been previously shown to provide a reliable estimate of the size of F-actin bundle structures in solution.^10,16,17^ Medians are indicated by orange lines, with boxplot whiskers marking the ±1.5× interquartile range. Outliers are indicated as empty circles above and below whiskers. Statistical significance of the difference between the measured intensity values was evaluated with the Mann–Whitney *U* test, with the markings corresponding to the following *p* values: n. s. (*p* ≥ 0.05), * (*p* < 0.05), ** (*p* < 0.01), and *** (*p* < 0.001). Black notations mark the significance levels between adjacent D_2_O concentrations, whereas gray notations compare the control measurement (0% D_2_O) with the three “plateau” values ((i) 20% and 30% D_2_O; (ii) 40%, 50% and 60% D_2_O; and (iii) 70% D_2_O).

In order to directly estimate the prevalence and size of bundles in solutions, we used static light scattering (SLS). We recorded the intensity of the scattered light from the solution containing F-actin networks in varying D_2_O buffer conditions. The networks were polymerized within a UV cuvette at the same concentration and in the identical manner as for the rheological investigations. Upon initiating the polymerization, the samples were left to equilibrate for 2 hours before proceeding with the SLS measurement. SLS measurements revealed a clear trend of increasing intensities with increasing D_2_O concentrations (Fig. 2), thereby providing a direct confirmation of the presence of bundle structures in networks. Only the scattering intensity of the sample containing 10% D_2_O was comparable to that of the control measurement (∼48 000 kcps), with samples with 20% and 30% D_2_O resulting in mean scattering intensity 1.3 times that of the control. In the concentration range of 40% to 60% D_2_O, the scattering intensity increases again but seems to plateau somewhat with a mean value roughly 1.9 times that of the control. Lastly, 70% D_2_O concentration featured the highest scattering intensity, roughly 2.5 times that of the control measurement.

### C Fluorescence microscopy

**Fig. 3:**
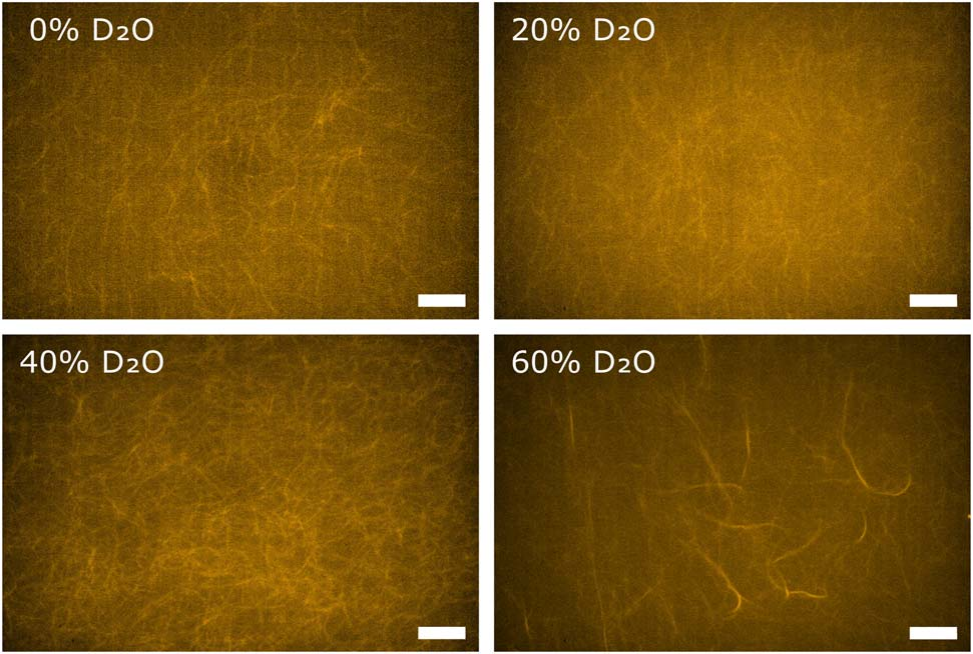
Fluorescence microscopy imaging confirms increasing anisotropy of F-actin networks with increasing heavy water content. Single frames, taken in the range between 0% and 20% D_2_O, networks appear mostly isotropic. With further addition of heavy water, networks display less ordered structure, with eventual bundle formation occurring at concentrations above 40% D_2_O. Scale bars correspond to 10 μm.

In order to obtain direct optical evidence of D_2_O induced polymorphisms in F-actin networks, we used fluorescence microscopy. Globular actin was polymerized to F-actin networks at actin concentrations of 0.04 mg ml^−1^ in buffers containing D_2_O in concentrations corresponding to those used for the rheological characterization. Using the same actin concentrations as for rheology and light scattering is impeded by the fact that individual filaments and bundle structures are not discernible in dense fluorescent labelled actin networks. Hence, fluorescence microscopy measurements were conducted at lower actin concentrations than rheology and light scattering, but can be considered as a visual evidence about the fundamental structural change in the F-actin networks. The reference F-actin network, polymerized in F-buffer containing no D_2_O, resulted in an entangled isotropic network (Fig. 3, 0% D_2_O), as expected. In the networks polymerized in buffer conditions containing D_2_O we observed the distinct formation of bundle structures embedded in the background of a percolated isotropic network. Generally, the prevalence of bundle structures increased with increasing D_2_O content (Fig. 3). These findings were in line with scattering intensities measured on respective networks *via* static light scattering, attributing the emergence of bundles to the effect of D_2_O.

## III. Discussion

Possible causes for the polymorphism of bundle formation in polymer networks are diverse. Actin binding proteins, ubiquitous in eukaryotic cells, induce a transition to anisotropic filament formations when present in sufficient concentrations.^17^ Lorenz *et al.* showed that the addition of crosslinkers to entangled F-actin solutions leads to the emergence of bundle formations above a certain threshold concentration of crosslinkers.^10^ Inter-filament actin association does not require specific binding sites but can be induced by a broad class of actin bundling factors. The application of depletion forces by the addition of depletion agents are a commonly used experimental technique to create multifilament bundles.^18^ The emergence of bundles in F-actin networks has also been observed as a consequence of the presence of polycations.^19,20^ Similar to bundling mechanisms involving crosslinkers and depletion agents like polyethylene glycol, a threshold concentration of polycation is required to form lateral aggregates of actin filaments. This threshold concentration is reflected in a sudden and steep increase in the measured scattering intensities above the respective threshold concentrations for crosslinkers,^10^ polycations^18^ or depletion agents.^19^ We show that the presence of D_2_O strengthens attractive filament–filament interactions and ultimately leads to bundling in entangled networks of filamentous actin. This constitutes an entirely novel actin bundling mechanism, which differs from those described so far. Employing multiple measurement methods, we provided strong evidence of bundle formation in a broad range of heavy water concentrations. We found scattering intensities that increase continuously with increasing D_2_O concentrations. This contradicts the existence of a hard and sudden D_2_O threshold concentration required for bundle formation and indicates that D_2_O-induced bundling features a broad coexistence regime of bundles and percolated network, spanning the entire range of D_2_O concentration points we measured. In contrast to previous studies, which reported strain-stiffening for networks of actin filaments, coupled *via* crosslinkers^21^ or depletion forces,^22^ we observed no such behavior in the nonlinear strain regime. The forces acting here thus seem to be strong enough to initiate a transition from a homogeneous entangled network to a network in which entangled filaments coexist with bundled actin filaments, but too weak to withstand strains in the nonlinear regime. Combining fluorescence microscopy and static light scattering, we conclusively related the formation of bundle structures solely to the effect of D_2_O on actin structures. Despite using considerably smaller actin concentrations for fluorescence microscopy, we observed the formation of bundles for increased D_2_O concentrations. Possible effects that arise from changed mixing dynamics due to physical properties of D_2_O and may interfere with the described polymorphism were experimentally excluded by thorough mixing of actin, buffer and D_2_O prior to the initiation of actin polymerization. We hypothesize that the origins of this novel bundling mechanism are in the D_2_O-mediated intensification of hydrophobic interactions. Increased hydrophobic interactions have previously been considered as the main cause for the observed structural tightening of several protein systems in the presence of heavy water.^23^ It is understood that filament assembly and complexing with many actin binding proteins rely on hydrophobic interactions.^24–26^ On the monomer level, hydrophobic interactions constitute a driving force in protein folding as hydrophobic parts of the globular protein inherently turn into the monomer's interior to prevent contact to its aqueous surrounding.^27^ Exchanging hydrogen bonds with stronger deuterium bonds may lead to the disclosure of the hydrophobic pockets within the amino acid sequence of actin molecules. Representing a relevant force on the scale of nanometers,^28^ the increased prevalence of hydrophobic patches along the contour of F-actin is likely to promote the formation of bundles in entangled networks. However, given the fact that actin molecules as well as their interaction with water are highly complex, this is one of several plausible explanations and it is likely that multiple mechanisms superimpose. The replacement of a proton by deuterium results in an alteration of the high-frequency dynamics of water. In addition to the rotational motion, the on–off diffusion between a protein bound state and a free state is changed in particular.^19^ Cells employ a variety of crosslinkers of different kind. Consequently, cytoskeletal polymers are largely present in the cell in a crosslinked state.^29^ Networks of α-synuclein stiffen significantly upon the disclosure of hydrophobic patches through a temperature increase.^29^ Being present in a crosslinked state prior to the treatment, they do not change their morphology but retain their architecture. Stronger filament–filament interactions cause a retardation in the mode relaxation dynamics, ultimately leading to stiffer networks. Likewise, it is expected, that cells show a monotonic stiffening behavior when subjected to increasing concentrations of D_2_O. The presented results provide an explanation for previously counter-intuitive non-monotonic viscoelastic responses, observed in the rheology of F-actin networks in the presence of heavy water.^5^ The formation of bundles is at the expense of the remaining isotropic percolated network. This explains the weakening of the overall network at intermediate D_2_O concentrations, reflected in higher loss factor values. High D_2_O concentrations lead to thicker and more stable bundles, overriding the effect of de-percolation. Consistent with previous studies, we explain our findings with a D_2_O mediated increase of hydrophobicity, however, we suspect that this is not the only mechanism that leads to the enhanced attractions between filaments. We found that these interactions are strong enough to cause the formation of anisotropic formations of actin filaments, constituting a so far unknown bundling mechanism.

## IV. Conclusion

Deuterium oxide enhances intermolecular forces most likely due to the intensification of hydrophobic interactions. Entangled networks of F-actin undergo a structural reorganization that results in the establishment of bundle formations. Crosslinked systems which better resemble the reality of the nature of cells may stiffen upon this treatment. In order to model the impact of D_2_O on cell systems on a subcellular level a crosslinked system composed of multiple different polymer model system can provide more detailed insights.

## Author contributions

P. M. and D. P. contributed equally to this work. P. M.: methodology, data curation, formal analysis, writing – original draft, review & editing; D. P.: methodology, data curation, formal analysis, writing – original draft, review & editing; T. B.: conceptualization, writing – review & editing; J. K.: conceptualization, supervision, resources, writing – review & editing; J. S.: conceptualization, methodology, supervision, resources, writing – review & editing.

## Conflicts of interest

There are no conflicts to declare.

## Supplementary Material

RA-013-D3RA03917J-s001
