# Supplementary material for: Heavy water induces bundling in entangled actin networks
Source: RSC Adv. 2023 Aug 18;13(35):24795–800. doi: 10.1039/d3ra03917j (PMC10437092; doi:10.1039/d3ra03917j)
Supplement: RA-013-D3RA03917J-s001 [file RA-013-D3RA03917J-s001.pdf]

Electronic Supplementary Material (ESI) for RSC Advances.
This journal is © The Royal Society of Chemistry 2023

Supplemental Material
Heavy water induces bundling in entangled actin networks

Paul Mollenkopf,[1] Dusan Prascevic,[2] Thomas M. Bayerl,[3] Josef A. Käs,[2] and Jörg Schnauß[2,4,5]

[1] Department of Physiology, University of Pennsylvania, Philadelphia, PA 19104, USA
[2] Peter-Debye Institute for Soft Matter Physics, Leipzig University, 04103 Leipzig, Germany.
[3] D2 Bioscience Group Ltd, Trinity Hall, 43 Cedar Ave., Hamilton HM LX, Bermuda.
[4] Fraunhofer Institute for Cell Therapy and Immunology, 04103 Leipzig, Germany.
[5] Unconventional Computing Lab, Department of Computer Science and Creative Technologies, University of the West of England, Bristol BS16 1QY, UK.

I. MATERIALS AND METHODS

A. Actin preparation

G-actin was prepared from rabbit muscle and stored at $-80\,°C$ in G-Buffer ($5\,mM$ TRIS HCl pH 7.8, $0.2\,mM$ ATP, $0.1\,mM$ $CaCl_2$, $1\,mM$ DTT, $0.01\,\%$ NaN_3), as described previously [1]. For experimental use, small volumes of monomeric actin were aliquoted and slowly thawed prior to the experiment. Fluorescently labeled actin was prepared by polymerizing G-actin at $5\,mM$ in a 1:1 ratio with phalloidin–tetramethylrhodamine B isothiocyanate (phalloidin–TRITC – Sigma-Aldrich Co.). Polymerization of actin samples was induced by adding 1/10 volume fraction of 10 times concentrated F-buffer ($5\,mM$ TRIS HCl pH 7.8, $1\,MKCl$, $10\,mM$ $MgCl_2$, $2\,mM$ ATP, $10\,mM$ DTT).

B. Rheology

Shear rheology measurements were performed with a dynamic shear rheometer (ARES, TA Instruments, USA or MCR 502, Anton Paar, Germany) and a cone–plate geometry with a diameter of $25\,mm$ and a gap width of $50\,\mu m$. Actin was polymerized between plate and cone for 2 hours at $20\,°C$ after initiating polymerization by adding 10-fold F-buffer and water to a final volume of $175\mu l$. To prevent interfacial elasticity artifacts, the cone was surrounded with a DMPC solution dissolved in dichloromethane at a concentration of $0.3\,\mu g/ml$. Following the application, DMPC assembles into a monolayer surrounding the geometry, thereby eliminating air exposure of the polymer solution. The sample chamber was sealed with a cap equipped with wet sponges to prevent evaporation. The measurement sequence consisted of following measurements (i-v): (i) Polymerization was monitored with a dynamic time sweep with one measurement point every $120\,s$ at a frequency of $f = 1\,Hz$ and a strain of $\gamma = 5\,\%$. Only those samples that were in equilibrium at the end of the time sweep were considered for further analysis. The linear regime was first measured with (ii) a short dynamic frequency sweep with a strain of $\gamma = 5\,\%$ in the range of $f = 0.01 - 10\,Hz$ with 7 points per decade, followed by (iii) a long dynamic frequency sweep with a strain of $\gamma = 5\,\%$ in the range of $f = 0.001 - 30\,Hz$ with 21 points per decade, before repeating the (iv) short dynamic frequency sweep again ($\gamma = 5\,\%$, $f = 0.1 - 10\,Hz$, 7 points per decade). Lastly, (v) a transient step rate test at a strain rate of $0.1\,s^{-1}$ was used to measure the non-linear strain regime. The differential shear modulus K was determined from the resulting stress–strain curves with a self-written Python script. K was calculated as the gradient of the spline fit smoothed stress data divided by the strain step width. The linear value K_{lin} was defined at the first non-negative stress value. Negative stress values, particularly for small strains, appear due to measurement limitations as well as a result of the fitting routine.

C. Static light scattering

Static light scattering (Malvern Instruments Ltd., Zetasizer Nano ZSP, UK) at a wavelength of $633\,nm$ was used to observe the dependence of actin network morphology on D_2O concentrations. The actin concentration was $12\,\mu M$. The D_2O concentration was increased in $10\,\%$ increments from $0\,\%$ to $70\,\%$. The scattering of the sample was measured every 15 seconds for $0.5\,h$ before scattering intensities were arithmetically averaged.

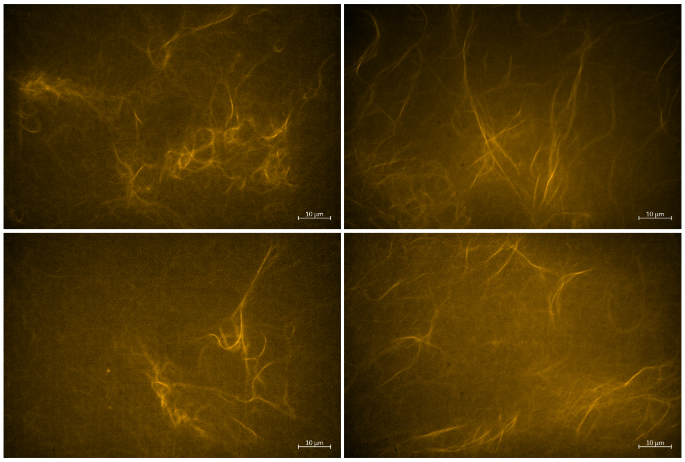

FIG. 1. Various bundle structures visualized by staining actin filaments with TRITC-Phalloidin at a concentration of $0.04\,mg/ml$ and $50\,\%$ D_2O relative concentration. Bundle structures appear to be embedded in a mostly isotropic background network of filaments.

D. Spinning disk confocal microscopy

For visualization, monomeric actin was mixed at a molar ratio of 1:1 with phalloidin tetramethylrhodamine isothiocyanate (phalloidin-TRITC) purchased from Sigma-Aldrich. D_2O was added to yield volume concentrations between $0\,\%$ D_2O and $70\,\%$ D_2O, in increments of $10\,\%$. After mixing all components, polymerization was initialized by adjusting the salt concentration to match 1x F-buffer conditions, with a final actin concentration of $0.04\,mg/ml$. Immediately after the polymerization process was started, the premixed solution was deposited into a sample chamber as described previously. Measurements were performed on a spinning disc confocal microscope (inverted Axio Observer.Z1/Yokogawa CSU-X1A 5000 (Carl Zeiss Microscopy GmbH, Germany), 100x oil immersion objective (Plan-Apochromat 100x/1.40 Oil DIC M27)) and recorded with a Hamamatsu camera at an exposure time of $100\,ms$. Fig. 1 shows microscopy images at a D_2O concentration of $50\,\%$.

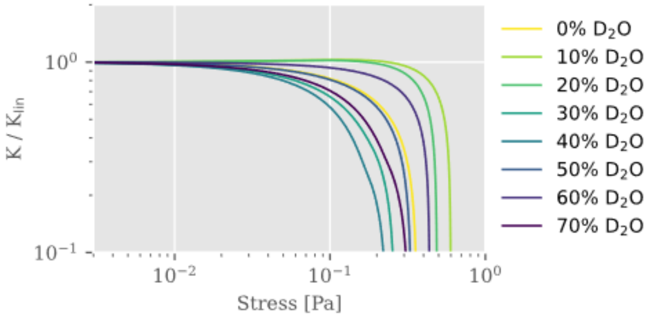

FIG. 2. Differential shear modulus normalized by its value in the linear regime K/K_{lin} as a function of measured stress. Based on the same data set as the curves shown in Figure 1d of the main manuscript, this representation allows for determination of the stress values at which networks yield (listed in Table 1). The values of yield stress do not directly correlate to the amount of heavy water present in the solution and exhibit non-monotonic behavior similar to other rheological parameters.

II. NONLINEAR RHEOLOGY

TABLE I. Yield stress values for varying heavy water concentrations, obtained by plotting K/K_{lin} as a function of stress σ (Fig. 1)

D_2O Concentration	Yield stress [Pa]
0%	0.363
10%	0.599
20%	0.488
30%	0.262
40%	0.232
50%	0.335
60%	0.438
70%	0.321

[1] B. Gentry, D. Smith, and J. Käs, Buckling-induced zebra stripe patterns in nematic f-actin, Physical Review E **79**, 031916 (2009).
